# Microarray analysis of host gene expression for comparison between naïve and HSV-1 latent rabbit trigeminal ganglia

**Published:** 2008-07-03

**Authors:** Christian Clement, Michael P. Popp, David C. Bloom, Gregory Schultz, Li Liu, Donna M. Neumann, Partha S. Bhattacharjee, James M. Hill

**Affiliations:** 1 Department of Ophthalmology, New Orleans, LA; 2 Departments of Pharmacology, Microbiology and Neuroscience Center, Louisiana State University Health Sciences Center, New Orleans, LA; 3 Department of Obstetrics and Gynecology, Gainesville, FL; 4 Department of Molecular Genetics and Microbiology, Gainesville, FL; 5 Department of Ophthalmology, University of Florida College of Medicine, Gainesville, FL; 6 University of Florida Interdisciplinary Center for Biotechnology Research, Gainesville, FL

## Abstract

**Purpose:**

To analyze the rabbit host global gene expression patterns in uninfected and herpes simplex virus type 1 (HSV-1) latent trigeminal ganglia (TG) for identification of host response-initiated transcriptional changes during the maintenance of viral latency.

**Methods:**

The corneas of eight-week-old New Zealand White rabbits were scarified and inoculated with HSV-1 strain McKrae, 5x10^5^ plaque forming units/eye. Corneal infection was verified by slit-lamp examination. Prior to sacrifice at 30 days post infection, ocular swabs confirmed no infectious virus was present. TG were aseptically removed from rabbits and placed in RNA stabilization solution. Host RNA was isolated from two groups of TG, uninfected and HSV-1 latent infected, and used to create labeled cRNA. Labeled cRNA was hybridized to two new and novel custom oligonucleotide rabbit arrays, containing a total of 3,123 probes for rabbit genes.

**Results:**

The rabbit TG expressed approximately 80% of genes out of a total of 3,123. A one-way ANOVA performed on the log2 transformed signal ratios showed 611 genes were significantly altered (p≤0.05) in HSV-1 latent TG. These genes, if annotated, were separated by biologic process categories. Five broad categories were most heavily represented: protein processing, carbohydrate processing, cell adhesion, apoptosis, and host defense and immune response. Sixty of the significantly altered genes were found to be altered by more than 2 fold, and five were altered by more than 4 fold. The genes altered by more than 4 fold were all upregulated and related to host defense and immune response. Viral latency had a large effect on protein processing. Of the differentially expressed genes with an assigned biologic process, 90/349 (25.7%) were associated with protein processing. The next most populated categories were carbohydrate metabolism 39/349 (11.1%) and host defense and immune response 17/349 (4.9%).

**Conclusions:**

The results of this microarray study demonstrate that host gene expression is altered in the HSV-1 latent rabbit TG. The shift in molecular processes at a pathway level reveals the presence of potential therapeutic significance inherent in the maintenance of HSV-1 latency. This is the first large-scale rabbit gene expression study, using microarray analysis, that documents the involvement of host immunity in maintaining HSV-1 latency.

## Introduction

A unique feature of herpes simplex virus (HSV) is its ability to establish a permanent latent infection for the life of the host [1,2]. During primary infection, HSV invades the neurons that innervate the mucosal surface by retrograde axonal transport to the neuronal nuclei that are housed in the sensory ganglia. In animal models, HSV-1 infection of surrounding neurons is observed after the virus replicates briefly in the neurons [3]. Latent HSV infection is then established in which functional viral genomes are retained in neuronal nuclei. HSV DNA persists in sensory ganglia of the peripheral nervous system [4] and is characterized by occasional reactivation of infectious virus. This could manifest as deadly brain inflammation or outbreaks of mucocutaneous lesions at peripheral sites innervated by the infected ganglia [5]. Reactivation may be spontaneous (non-induced) or triggered, effected mainly by host factors, including altered expression of cellular components such as induction of transcriptional activators and downregulation of repressors. Factors such as hormonal changes, stress, and ultraviolet (UV) exposure can induce the reactivation of the latent virus [6-9]. Reactivation can also be triggered by systemic changes in immune modulators, physical probing, and immunological or pharmacological induction of neuronal and surrounding tissue [10-18]. Viral reactivation has been triggered by stressors including epinephrine iontophoresis [15], timolol [19], and nicotine [16] in rabbits, and cyclophosphamide and dexamethasone immunosuppression-induction [10,13], sodium butyrate [17], and cadmium [20] in mice.

During latency, expression of viral genes is suppressed, with the exception of latency-associated transcripts that are the only abundantly present viral transcripts in latent infected neurons [21]. Latency, thus, has been described as a “passive phenomenon” due to lack of activation of the acute phase of viral pathogenesis [21]. In contrast, early molecular events during reactivation from latency suggest a more dynamic transcriptional activity [18,22]. However, molecular mechanisms in neuronal cells that regulate transformation from acute infection to latency and influence the ability of the virus to reactivate remain under investigation. There are also species differences underlying reactivation. The virus undergoes spontaneous, episodic reactivation with or without evidence of recurrent disease in humans and rabbits. In contrast, mice either do not undergo spontaneous reactivation or undergo spontaneous reactivation at such a low frequency or low amount that it is difficult to detect [23].

The pathways of HSV-1 acute phase or lytic infection are well understood. Although the contribution of the host immune system to maintaining the viral genome in a latent state is known, the actual processes resulting in the establishment, maintenance, and reactivation from latency are largely unclear [24]. Microarray analysis of host gene expression in response to viral infection is increasingly being used to more comprehensively assess genetic function and cellular response. Altered host gene expression has been demonstrated with varying patterns of expression evident with increasing time following hyperthermia of latent infected mice [22,25].

HSV infects the majority of the human population [26]. A high rate of viral shedding is prevalent as 98% of 50 human subjects studied over 30 days have been reported to have each shed HSV-1 viral DNA at least once over this time period [27]. Thus, there is a high transmission rate and prevalence of the virus in the world population [24]. In most individuals the virus appears to be “immobilized” in the latent state. However, in some individuals outbreaks are frequent and probably parallel molecular and cellular changes geared toward suppressing the reactivation of the virus [24]. To understand pathways with potential therapeutic implications that influence viral latency, we infected New Zealand White (NZW) rabbits with HSV-1 under conditions that favor latency for comparison to uninfected naïve controls. After the rabbits were sacrificed, the trigeminal ganglia (TG) were aseptically removed and rapidly processed for transcripts. The transcripts were used to elucidate gene expression during latency.

## Methods

### Rabbits, viruses, infection, and removal of trigeminal ganglia

All animal procedures followed the “Principles of Laboratory Animal Care” (NIH publication No. 86–23, revised 1985) using a protocol approved by the LSU Health Sciences Center Institutional Animal Care and Use Committee. Ten eight-week-old NZW rabbits (Black Creek Rabbitry, Moss Point, MS) were conventionally housed (singly) in an Association for Assessment and Accreditation of Laboratory Animal Care accredited facility in stainless steel suburban surgical cages under a 12:12 light dark cycle. Humidity was maintained between 30 and 70% and temperature at 61–72 ^ο^F in a negatively pressured room with 10–15 air changes per hour. The rabbits were given Harlan global 2031 rabbit food and water ad libitum. The rabbits were anesthetized by intramuscular injection using 50 mg/kg ketamine hydrochloride and 10 mg/kg xylazine (Vedco Inc., St. Joseph, MD). The corneas were scarified and inoculated with 25 µl HSV-1 strain McKrae, 5x10^5^ plaque forming units (pfu)/eye, or PBS for uninfected (naïve) controls. Slit-lamp examinations and ocular swabs were performed two and three days after viral inoculations to verify 100% corneal infection. To verify that latency had been established at 30 days postinoculation, we cocultured ocular swabs and primary rabbit kidney cells. All were found negative for infectious virus. Rabbits were anesthetized as described and then euthanized with 100 mg/kg sodium pentobarbital by intracardiac injection. The crania were opened, and TG aseptically removed. The TG were stored in RNAlater® (Ambion, Austin, TX) at −80 °C until ready for use.

**Table 1:** Primer sequence for real-time PCR analysis

Gene	Primers (5’-3’)
*IGJ*	F: TGAAGAGCCATTTGCTTCTCTGGG
	R: ATGATCCTGGAAGTGATGCGGACA
*IL1β*	F: AGCTCACCAGTGAGATGATGGCTT
	R: TCATGTAATTGGGACCATCGGCCT
*LYZ*	F: AGAGGTCTTGCTTCCCAGTCAACA
	R: ACTCCCTTGTAGCCATCCAATCCA
*CAP18*	F: ACTTCAAGGAAGATGGGCTGGTGA
	R: TGTTGCAGCGGATGTCAAAGGAGT
*CRISP3*	F: GCCCTGATCACTGTGACAATGGACTA
	R: CAGCTGGCCTTGCAGTTGTTCTTA
*RPL27A*	F: ACACGGGTAAATGCTGCCAAGAAC
	R: TTGGGAGCTTTCCCTTTCCCAGAA
*GAPDH*	F: GAACATCATCCCTGCATCCA
	R: CCAGTGAGCTTCCCGTTCA

### RNA Isolation and preparation of fluorescently labeled cRNA

Total RNA was extracted from TG using an RNeasy® kit (Qiagen Sciences, Germantown, MD) and submitted to the University of Florida’s Interdisciplinary Center for Biotechnology Research Gene Expression Core Facility (Gainesville, FL). Prior to target labeling, the purity of each sample was evaluated using a 200 ng aliquot with a 2100 Bioanalyzer (Agilent Technologies, Palo Alto, CA). Purity was assessed based on the relative abundance of the 18S and 28S ribosomal bands and on the presence of baseline rise, both of which reveal RNA degradation. Samples were prepared for hybridization according to the Low RNA Input Fluorescent Linear Amplification Kit protocol (Agilent Technologies).

A 300 ng aliquot of total RNA was used as a template for cDNA synthesis. The subsequent product served as a template for in vitro transcription, during which one of two cyanine-labeled nucleotides (Perkin Elmer, Wellesley, MA) was incorporated into the synthesized cRNA. All eight experimental RNA samples were labeled with cyanine 5-cytosine triphosphate. A reference RNA sample was prepared by pooling an aliquot from each of the eight samples. The reference RNA was labeled with cyanine 3-cytosine triphosphate. In vitro transcription reactions were cleaned with RNeasy® Mini columns (Qiagen, Valencia, CA), and cRNA concentration and specific activity were both measured with a ND-1000 spectrophotometer (NanoDrop Technologies, Wilmington, DE). The quality of each cRNA sample was evaluated with emphasis on total yield, specific activity of product, and by the presence of a smooth, mRNA-shaped curve of an appropriate range in fragment size.

**Table 2:** Summary of genes with an assigned Gene Ontology term whose expression was significantly altered in the HSV-1 latent trigeminal ganglia

Upregulated (total 20)	x=fold change			Down-regulated (total 40)	x=fold change		
	4x	2x	<2x		4x	2x	<2x
Immune response and defense	5	3	5	Protein degradation	0	0	9
Protein biosynthesis	0	1	3	Protein biosynthesis	0	0	4
Glycolysis	0	0	1	Carbohydrate metabolism	0	5	18
Apoptosis	0	0	2	Apoptosis	0	0	4

The total number of genes whose expression was altered in the HSV-1 latent trigeminal ganglia comprised genes that were upregulated and genes that were downregulated. HSV-1 latency overall favored downregulation of genes in the catabolic categories of protein degradation and carbohydrate metabolism.

### Array hybridization and generation of expression values

Hybridization cocktails were prepared for each sample by combining a 100 ng aliquot of the cyanine 5-cytosine triphosphate-labeled experimental sample and a 100 ng aliquot of the cyanine 3-cytosine triphosphate-labeled reference RNA with a high salt buffer. This mixture was incubated at 60 °C for 30 min to fragment the labeled cRNA into 30–200 base strands. The fragmented reaction mixture was used to hybridize two new and novel custom oligonucleotide rabbit arrays (Agilent Technologies) manufactured in the eight-pack format (eight individually hybridizable 1.5K arrays per piece of glass). A representation of the array plates is shown in Appendix 1. Arrays were hybridized at 60 °C for 17 h in a rotating oven and passed through both a low and a high stringency wash according to the manufacturer’s protocols, dried with filtered nitrogen gas, and scanned for each of two wavelengths (green: 570, red: 670) with a G2505 B Scanner (Agilent Technologies). Signal values were adjusted to account for both local background and potential differences in hybridization intensity across the array. Signals not significantly above background were considered negative. Because accurate estimates of signal ratios cannot be calculated if neither the red nor green signal is above background, a gene was considered present (expressed under experimental conditions) only if both the red and green signals from its representative probe were above background on each array. If considered present, a log2 red:green signal ratio was calculated individually for each probe on each array. Signal ratios were normalized and used for analysis of gene expression. Probes for housekeeping genes *GAPDH* and β-actin (*ACTB*) were included on the arrays as internal controls. Signal acquisition, evaluation, and transformation were performed with Feature (Cap E) Extraction software version 8.1 (Agilent Technologies).

### Analysis of expression data

To determine the effect of HSV-1 latency on gene expression, we performed a one-way ANOVA on the log2 transformed red:green ratios for two treatments (naïve TG and HSV-1 latent TG) and four replicates. Statistical tests were performed with AnalyzeIt Tools.

For each unique contig, a homology search was performed using the BLASTX and BLASTN application against the NCBI NR and NT databases, respectively. BLAST results were parsed and stored in BlastQuest, a SQL database developed by ICBR that facilitates the management of BLAST results and Gene Ontology (GO) Consortium assigned biologic processes (Ophthalmic Gene Microarray Project) [28]. Although each probe represents a unique sequence, some genes are represented by multiple probes. For the purpose of this manuscript each probe is referred to as a gene.

**Figure 1:**
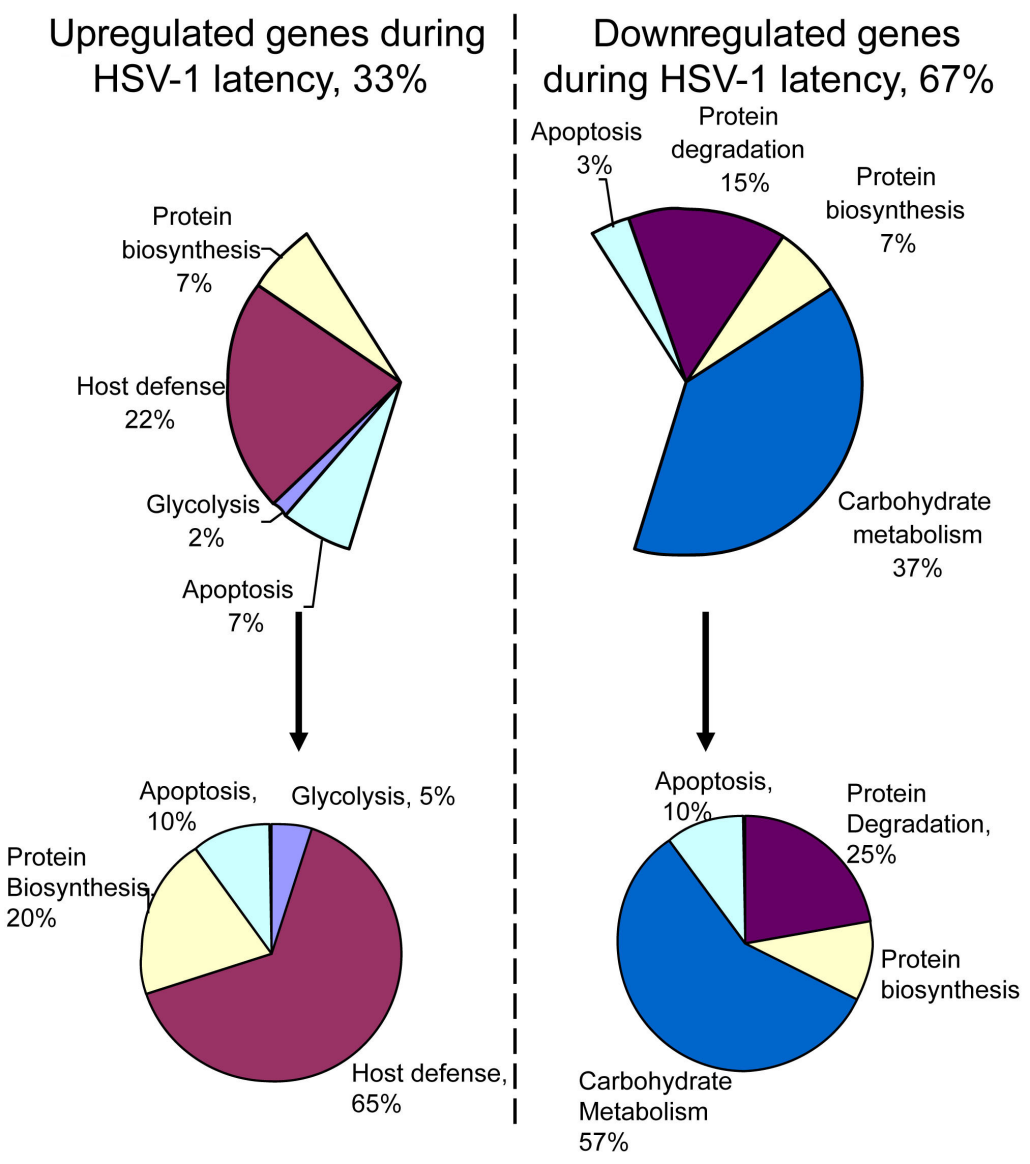
Gene expression significantly altered in the rabbit HSV-1 latent trigeminal ganglia compared to naïve (uninfected) trigeminal ganglia. Pie chart of gene expression significantly altered in the HSV-1 latent rabbit trigeminal ganglia compared to naïve trigeminal ganglia. Host defense and immune response genes showed the highest percentage of altered genes that were upregulated. The catabolic categories of protein degradation and carbohydrate metabolism showed the highest percentage of altered genes that were downregulated.

### Quantitative real-time polymerase chain reaction

Gene expression levels for the microarrays were validated by quantitative real-time polymerase chain reaction (real-time PCR). The real-time PCR analysis was done on HSV-1 latent rabbit TG and naïve TG RNA samples for six rabbit genes shown in Table 1. Real-time PCR was concurrently done for rabbit *GAPDH* and its expression served as an internal control. All primer pairs for the six genes were specifically designed and synthesized by Integrated DNA Technologies, (San Diego, CA). Reverse transcription was primed with T7-Oligo (dT) containing primer (Affymetrix Inc., Santa Clara, CA). Real-time PCR was performed in a 20 µl volume containing a solution of 1X DyNAmo Flash SYBR® green Master Mix (Finzymes, Keilaranta, Espoo, Finland), 0.5 µM forward primer, 0.5 µM reverse primer, and 1 µl cDNA or 20 ng reverse transcribed total RNA. The reaction was run using a four-step protocol: denaturation, 10 min at 95 °C; amplification and quantification, 40 cycles at 15 s for 95 °C and at 1 min for 60 °C; melting curve, 60–95 °C with a heating rate of 0.5 °C/s followed by cooling. The real-time reactions were done using MyiQ™ Single Color Real-Time PCR detection System (Bio-Rad, Hercules, CA). Amplicons of the real-time PCR were analyzed using 2% agarose gel electrophoresis and each product separated into a single sharp band of the correct size. HSV-1 latent rabbit TG and naïve TG samples were assayed in triplicate. Relative quantitative expression levels were determined for each gene. All results are expressed as an expression ratio of the HSV-1 latent TG to naïve TG, normalized against *GAPDH* expression levels using the 2^-ΔΔ^*^C^*_T_ method [29].

## Results

Of the 3,123 genes on the two new and novel custom oligonucleotide rabbit arrays (Agilent Technologies), 2,509 genes were expressed in our experimental conditions. A one-way ANOVA was performed on the log2 transformed signal ratios which identified 611 genes whose expression was significantly altered (p≤0.05) in HSV-1 latent rabbit TG (Appendix 2 and Appendix 3). The null hypothesis was rejected if the ratio of the naïve and HSV-1 latent treatment means was significantly different. When this list of genes was further filtered on the basis of fold change, it was found that the expression of 60 genes differed by more than 2 fold and a subset within these 60 genes changed by more than 4 fold (Table 2, Appendix 4, and Figure 1). Real-time PCR done for six selected genes within these 60 genes mirrored a pattern of fold change consistent with those of the arrays (Appendix 5). Of these 60 genes, the expression of 20 increased (Table 3) and 40 decreased (Appendix 6) relative to naïve tissue. The genes whose expression changed by more than 4 fold were a subset comprising five genes belonging to the 20 genes whose expression increased (Appendix 2 and Table 4). These five upregulated genes all had an assigned GO term of host defense and immune response: *IL1β*, *CRISP3*, *CAP18*, lysozyme (*LYZ*), and immunoglobulin joining chain (*IGJ*). Two more genes within this group of 20 also had an assigned GO term related to host defense and immune response: major histocompatibility complex class II DQ alpha 1 (*HLA-DQA1*) and amyloid A protein (*SAA*). An additional two genes also belonging to these 20 genes, human chemokine ligand 12 (*CXCL12*), and antibody variable domain, could be assumed to be involved in host defense and immune response based on their gene nomenclature. In contrast, within the group of 40 downregulated genes (Appendix 6) there was no obvious pattern in gene expression that would explain the maintenance of viral latency. Figure 1 is a graphic representation of the relative abundance of gene expression significantly altered in the rabbit HSV-1 latent TG compared to naïve TG. Figure 1 shows that the majority of the genes with upregulated expression are related to host defense and immune response, while the majority of those with downregulated expression are involved with carbohydrate metabolism.

**Table 3:** Expression of 20 genes significantly upregulated in the rabbit HSV-1 latent trigeminal ganglia

Gene name	Fold change
**Immune response and defense**	
Immunoglobulin Joining Chain (*IGJ*)	14.22
Human Lysozyme (*LYZ*)	8.63
Cathelicidin Antimicrobial Peptide (*CAMP*)	6.06
Cysteine-rich Secretory Protein 3 (*CRISP3*)	4.23
Interleukin 1, Beta (*IL1β*)	4.2
Amyloid A Protein DSAA85 Precursor (*SAA*)	2.56
Major Histocompatibility Complex, Class II, DQ Alpha (*HLA-DQA1*)	2.89
Antibody Variable Domain-like 1	2.27
Antibody Variable Domain-like 2	1.99
Antibody Variable Domain-like 3	1.78
Antibody Variable Domain-like 4	1.45
Interleukin 1 Receptor Antagonist (*IL1RA*)	1.77
Ceruloplasmin (*CP*)	1.68
**Protein biosynthesis**	
Ribosomal Protein L27a (*RPL27A*)	2.06
Ribosomal Protein S16 (*RPS16*)	1.59
Eukaryotic Translation Initiation Factor 3, Subunit 5 (*EIF3F*)	1.67
Eukaryotic Translation Initiation Factor 4A, Isoform (*EIF4A1*)	1.75
**Glycolysis**	
Pyruvate Dehydrogenase E1 Component Beta Subunit, Mitochondrial Precursor (*PDHE1B*)	1.13
**Apoptosis**	
Thioredoxin Domain Containing (*TXNDC*)	1.85
Amyloid Beta Precursor Protein (*APLP2*)	1.4

Gene expression was significantly (p≤0.05) altered by more than twofold in the HSV-1 latent rabbit trigeminal ganglia (TG). Twenty expressed genes were significantly upregulated in the rabbit HSV-1 latent TG of which a subset of five genes in the immune response and defense category were upregulated by more than 4 fold. Immune response and defense gene expression showed the highest upregulation in the rabbit HSV-1 latent TG.

**Table 4:** Subset of genes whose expression was altered more than 4 fold in the rabbit HSV-1 latent trigeminal ganglia

Probe name	p-value	Mean log2 difference	Fold change	Hit definition	Biologic Process	E-value
UF_Oc_c_40190	0.001	3.83	14.22	Immunoglobulin Joining Chain	Humoral immune response	0
UF_Oc_c_40194	0.004	3.11	8.63	Human Lysozyme	Cell wall catabolism; carbohydrate metabolism; cytolysis	0
UF_Oc_n_41508	0.032	2.6	6.06	Cathelicidin Antimicrobial Peptide	“Response to pest, pathogen or parasite; defense response to bacteria”	0
UF_Oc_c_40416	0.033	2.08	4.23	Cysteine-rich Secretory Protein 3	Fertilization; innate immune response; cell-cell adhesion; spermatogenesis; defense response	0
UF_Oc_n_41565	0.021	2.07	4.2	Interleukin 1 Beta	Apoptosis; cell proliferation; negative regulation of cell proliferation; signal transduction; inflammatory response; regulation of cell cycle; antimicrobial humoral response	0

The genes whose expression was altered by more than 4 fold were all upregulated and had an assigned Gene Ontology term related to host defense and immune response. These genes, interleukin 1 beta (*IL1β*), cysteine-rich secretory protein 3 (*CRISP3*), cathelicidin antimicrobial peptide (*CAP18*), human lysozyme (*LYZ*), and immunoglobulin joining chain (*IGJ*), are involved in innate and immune defense response.

To gain a more global perspective and potentially identify metabolic shifts ongoing during HSV-1 latency, we filtered all the differentially expressed genes by their GO terms. Only 349 of the genes had an assigned GO term for biologic process. The remaining 262 genes did not at the time of this study. When separated by ontology, five heavily populated biologic processes were identified: protein processing, carbohydrate processing, cell adhesion, apoptosis, and host defense and immune response (Appendix 3). Each category was further broken down into subcategories based on more specific GO terms. Thus, the protein processing group included genes representing biosynthesis, folding, modification, transport, and proteolysis and peptidolysis (Appendix 2). Viral latency had a large effect on protein processing which contained 90/349 (25.7%) of the differentially expressed genes with an assigned biologic process ontology. The next most populated categories were carbohydrate metabolism (39/349, 11.1%) and host defense and immune response (17/349, 4.9%). Examination of fold change values within ontology groups, in some cases, identified shifts in metabolism. For example, of the 2,509 genes that were considered expressed in our study, 121 represented genes involved in carbohydrate metabolism and energy production (Appendix 7). Thirty-nine of these genes were significantly affected by latency. Interestingly, 33 of the 39 were downregulated. Conversely, within the protein biosynthesis group, 27 of the 33 expressed genes significantly affected by latency were upregulated. Expression of all 23 genes associated with ribosomal proteins was upregulated. Peripheral to the major shifts, 12 genes associated with protein degradation were upregulated and five were downregulated. Forty-two genes involved in apoptosis were expressed, of which 11 were significantly altered, including seven genes that were upregulated. Forty-three genes involved in cell adhesion were expressed of which 11 were significantly altered including only three genes that were upregulated (Appendix 7).

Gene expression levels were validated by performing real-time PCR on six selected genes. The changes in expression levels of *IGJ*, *LYZ*, ribosomal protein L27a (*RPL27A*), *CRISP3*, *CAP18*, and *IL1β* are listed in Appendix 5. Of these six, the expression of only *IGJ* increased dramatically—that is, greater than 40 fold. The increase in expression of *RPL27A,* and *CRISP3* of about 2 and 4 fold for each gene, respectively, were almost identical in comparison to the array-based expression changes. While the expression of *LYZ*, *CAP18*, and *IL1β* increased moderately (about 3 fold), this was comparably lower than the array-based expression changes. Generally, the array-based expression changes were consistent with those of the real-time PCR in that the expression of all six selected genes significantly increased, and the trend of increment was maintained between the two methods of detection. Thus, it follows that of the 3,123 genes present on the arrays, *IGJ* had the highest increase in gene expression, and this trend corresponds to the high value of the real-time PCR. The values for the fold-change in expression levels of *RPL27A* and *CRISP3* for both the microarray and real-time PCR were basically the same. Further, *LYZ*, *CAP18*, and *IL1β* expression levels were all upregulated, but to a lower extent in the real-time PCR than the microarray.

## Discussion

This study used two new custom oligonucleotide arrays [28] to evaluate gene expression in the TG of rabbits harboring latent HSV-1. Of the 3,123 genes on the two arrays, the expression of 611 genes was altered during HSV-1 latency when compared to the gene expression in the uninfected rabbit TG.

During latency, the viral genome is stably maintained and viral transcription is limited to the expression of latency-associated transcripts. T-cell-secreted cytokines are believed to contribute to the effective establishment of latency by inducing host defense and immune response [30]. Considering the supposed quiescent state of the TG during latency, there is a relatively large proportion (approximately 20%) of genes represented on the arrays that were differentially expressed. This suggests a metabolically active interaction between the functional viral genome and the TG harboring latent HSV-1 DNA. The interaction most likely to have a direct effect on the virus is that dealing with host defense and immune response. The commitment of the host TG to defense is indicated by the percentage of defense and immune genes whose expression was significantly increased and by the magnitude of the increase. Of the genes that were most affected during latency, 20 were upregulated (Table 2, Figure 1). Thirteen of these 20, including all five that changed by more than 4 fold, are related to host defense and immune response.

The first line of host defense and immune response is innate and initiated by tissue damage. *IL1β*, which increased 4 fold, is a proinflammatory cytokine whose expression is triggered by tissue injury or infection. *IL1β* stimulates and mediates the hepatic synthesis and subsequent release of acute-phase proteins into the blood stream [31-33]. The function of the acute-phase proteins is systemic, resulting in the restoration of homeostasis, neutralization of pathogens, and promotion of conditions necessary for tissue repair [31-34]. The expression of three acute-phase proteins, ceruloplasmin (*CP*), interleukin 1 receptor antagonist (*IL1-RN*) and serum amyloid A protein (*SAA*) [32] was significantly increased during latency (Appendix 2). *CP*, whose expression increased by approximately 1.5 fold (68%) is believed to act as an anti-inflammatory and an immunomodulatory agent. The extrahepatic expression of CP is hypothesized to reduce inflammation-induced tissue damage [33]. *IL1-RN*, which increased by 1.75 fold (77%), binds to IL1 receptors and inhibits the binding of both IL1-α and IL1β. As a consequence, the biologic activity of these two cytokines is neutralized in immune and inflammatory responses [35]. Also, *CP* is an amyloid precursor protein (*APP*) gene. *APP* belongs to a family of conserved type I transmembrane proteins. This family includes amyloid beta precursor-like protein 2 (*APLP2*) [36-38]. The expression of *APLP2* increased in the HSV-1 latent rabbit TG (Appendix 2). *SAA* protein, which increased by approximately 2.5 fold, is most similar to the serum amyloid A3 (*SAA3*) isoform, which is the predominant extrahepatic form in rats, mice, and rabbits [31,39]. SAA3 is required for effective stimulation of collagenase, an extracellular matrix repair enzyme, by IL1β in rabbit corneal fibroblasts [40]. Tumor necrosis factor-alpha (*TNF-α*), a proinflammatory cytokine which is an important component of the innate response, was not detected. However, its modulator, TNF-αIP3 interacting protein 1 (*TNIP1*) decreased in the TG harboring latent HSV-1 (Appendix 2). TNIP1 interacts with transcription factor TNF-αIP3 with an attributed antiapoptotic role [41,42]. HSV-1 may induce this antiapoptotic pathway to paralyze any deleterious effects of IL1β.

In addition to acute-phase proteins, the expression of other innate defense-related genes was significantly increased during HSV-1 latency. *CRISP3* expression increased approximately 4 fold (Table 3 and Appendix 4). Although its function is not well established, the CRISP3 protein is localized together with lysozyme, collagenase, and gelatinase in human neutrophils. *CRISP3* is also found in exocrine secretions and is a major secretory product of macrophages, indicating a role in innate immunity [43-48]. *CAP18* is a histatin. CAP18 is produced by epithelial cells. These cells constitute the first line of defense against pathogens [49]. CAP18 has been demonstrated to bind to and neutralize lipopolysaccharide and lipoteichoic acid, both of which are considered to be mediators of inflammation [50,51]. Additionally, CAP18 is a chemotactic factor for T cells and dendritic cells. Lysozyme C (*LYZ*), which increased by 8 fold (Table 3 and Appendix 4), is a proteolytic enzyme that is a component of granules of neutrophils and is the major secretory product of macrophages. LYZ is thought to be directly involved in the defense against bacteria [43,44] due to its ability to hydrolyze the constituent polysaccharides of bacterial cell walls. *CRISP3* and *LYZ* are implicated in neutralizing pathogens, and all these immune gene expressions were significantly increased in the HSV-1 latent rabbit TG.

The immune defense response is the second line of defense against pathogenic challenge. As was seen with the innate host response, the commitment of the host to immunity was indicated by the percentage of defense genes whose expression was significantly increased and by the magnitude of the increase. Immunoglobulin joining chain was the gene whose expression changed the most, approximately 14 fold (Table 3). Immunoglobulin joining chain plays an important role in polymerization and secretion of IgA, pentameric IgM, and polymeric IgS [52,53], all of which are important protection from exposure to microbes in circulating body fluids. Other immunity genes that were significantly increased included a major histocompatibility complex gene and a gene for the antibody variable domain for which there were four individual genes (Appendix 2 and Appendix 4). The 14 fold increase of the immunoglobulin joining chain gene expression, together with the high percentage increase in expression of other host defense and immune response genes, indicates that the host immunity is activated to counter viral infiltration. HSV-1, to evade this heightened surveillance, probably imposes latency. Thus, the virus reverts to functional viral genome that is retained in neuronal nuclei in an inactive state to prevent its elimination by the host immune system. Alternatively, HSV-1 circumvents the host immune response by adapting to host immune mechanisms that seek to eliminate the virus [54].

Although the host defense and immune response functional group was the largest represented by genes whose pattern of expression changed, shifts in other metabolic pathways were observed. For example, of the protein biosynthesis genes considered expressed, 33/152 (21.7%) were significantly affected by HSV-1 latency. Within this group, all 23 genes associated with ribosomal proteins, including the subset of four genes associated with ribosome biogenesis, were upregulated (Appendix 7). Ribosome biogenesis is a complex, highly coordinated and energy intensive process. Because ribosome biogenesis is a major consumer of the cell’s resources, upregulation of ribosome biogenesis only occurs when extracellular conditions are favorable for rapid growth. This information is signaled through and regulated by the target of rapamycin (TOR) protein family. This family of proteins is highly conserved within eukaryotes and has a COOH-terminal kinase domain that phosphorylates serine and threonine residues [55,56]. Inhibition of TOR signaling by rapamycin causes global repression in the expression of genes coding for cytoplasmic ribosome proteins [57].

In addition to ribosome biogenesis, TOR signaling controls several other processes that are important for cell growth [56]. These processes include transcription, protein kinase C signaling, protein synthesis, protein degradation, membrane trafficking, and organization of the actin cytoskeleton. Thus, by regulating these processes, TOR signaling appears to control the balance between protein synthesis and degradation in concert with extracellular conditions [56]. In this study, we found that, for the significantly altered expression of genes, 23/23 (100%) of the genes coding for ribosomal proteins were upregulated, suggesting favorable conditions for growth (Appendix 2 and Appendix 7). Consistent with this idea, we found 12/17 (70.6%) of the differentially expressed genes coding for protein degradation decreased (Appendix 2 and Appendix 7). In conjunction, we found that many of the expressed genes that were associated with protein processing, transport 10/33 (30.3%), folding 15/38 (39.5%) and modification 15/69 (21.7%), were significantly altered (Appendix 7). All these results suggest a coordinated enhancement of the mechanism involved in cell growth.

Contrary to the direction of shifts observed in protein processing, we found an opposing shift in carbohydrate processing. Thirty-nine of the 121 genes representing genes involved in carbohydrate metabolism were significantly affected by HSV-1 latency. The majority, 33 of the 39, were downregulated. This is remarkable considering that the biogenesis of ribosomes is energy expensive and upregulation of all ribosomal protein transcripts suggests favorable growth conditions.

There was also a shift in the pattern of expression of apoptosis related genes. Of the 42 genes associated with apoptosis that were expressed, seven of the 11 genes significantly altered by HSV-1 latency were upregulated (Appendix 2 and Appendix 7). This seems peripheral to the observed shift in protein metabolism. However, apoptosis is also a process that maintains a balance between synthesis and degradation. Changes in apoptosis, affecting processes such as proteolysis and transport, and components of the apoptotic cell could possibly be recycled.

A shift in expression of genes related to cell adhesion was also observed. Only three of the 43 expressed genes associated with cell adhesion were upregulated (Appendix 7), although cell adhesion activity is important for interactions that allow viruses and bacteria to cause damage to tissues. Fibronectin (*FN*) gene expression was decreased significantly (Appendix 2). FN is involved in many cellular processes, including tissue repair, embryogenesis, blood clotting, cell migration and cell adhesion. A major function of FN is in the adhesion of cells to extracellular matrix materials, particularly collagen, and its presence is crucial in tissue repair [58]. Since the alterations in gene expressions of procollagen-Type XII alpha 1 (*Col12a1*) gene, which increased (Appendix 4), and *FN*, which decreased, were antagonistic, healing of tissue could be impaired—a condition that may be host-induced to delay viral spread.

Global gene expression assays using microarrays are becoming standard techniques to study the cellular and molecular response of the host cell during HSV reactivation. However, this study is unique as an analogous study has analyzed host gene expression in the HSV-1 latent mouse TG and during explantation [59]. This is the first rabbit study that has used this approach to measure changes that may occur during HSV-1 viral latency. Rabbits have been extensively used as a model for HSV latency and reactivation in TG [60,61]. However, the lack of a commercially available rabbit array has hampered the implementation of global gene expression research in rabbits. This study attempts to illuminate the key molecular events that either maintain latency or are used during the suppression of viral genes. No other animal study has focused exclusively on HSV-1 latent infection by microarray technology. This result is complementary to previous studies on array analysis of HSV-1 latent infected mouse TG [62], host gene response during viral reactivation [22,63], and cyclophosphamide and dexamethasone immunosuppression-induction reactivation of HSV-1 [13].

The results of this study indicate that latency involves a transcriptionally active response by the host. The most notable response of the host during latency is a metabolic shift toward protein synthesis, mediated by global upregulation of transcripts for ribosomal proteins, and a reduction in the transcript level of genes involved in protein degradation. Many genes coding for other proteins involved in protein processing, (i.e., protein transport, protein modification, and protein folding) were significantly altered. Of particular interest is the fact that all gene expression changes by at least fourfold were upregulated and related to immune response and defense. This also suggests an active role of the host in maintaining latency. This could also be brought about by lack of key transcription activation factor(s). It has been demonstrated that a complex interplay of virus, neuron and host immune surveillance is required to maintain latency [64].

Our selection of six genes (*IGJ, LYZ, RPL27A*, *CRISP3*, *CAP18*, and *IL1β*) for performing real-time PCR, was made because of the importance of the immune response and defense genes in this study. Five of these six genes, with the exception being *RPL27A*, belong to the defense category. In validating the microarray data, the real-time PCR data also provided confirmation that the immune response and defense gene expression changes associated with the HSV-1 latent rabbit TG are significant. The consistency and trend of upregulation of each gene obtained by these two methods of detection for all the six selected genes provide evidence of significant HSV-1 latency-associated alteration of specific genes out of the total 3,123 genes present on the arrays. Although some of the levels were different between these two methods of detection, we still observed the same trend of upregulation of each gene. Comparison of the ratio of fold-change values of gene expressions between the array and the real-time PCR showed differences only in magnitudes with some relatively minor: *IGJ*, +14.22:+44.32; *LYZ*, +8.63:+3.14; *RPL27A*, +2.06:+1.93; *CRISP3*, +4.23:+4.38; *CAP18*, +6.06:+3.05; and *IL1β*, +4.20:+2.68. This suggests that, overall, the microarray data were accurate. In support of our observation, previous studies [13,59,65-69] have reported changes in gene expression obtained from microarray analysis as usually similar to, but not always identical with, those obtained from real-time PCR [13,59,65,66]. Immunoglobulin joining chain is important in the protection from exposure to foreign antigens, and it is unclear whether its strikingly high increase in expression during latency is a direct viral manipulation or host induced response. However, this phenomenon could be an indicator of the immune element’s role in controlling the appearance of substantial viral proteins that may lead to the initiation of the reactivation phase.

Overall, our microarray study, in which the expression of genes associated with the host immune response and defense were increased by more than 4 fold, indicates that the immune system plays a significant role during the maintenance of HSV-1 latency. The alterations of molecular processes in which protein synthesis was upregulated as well as increases to major ribosomal proteins suggest the presence of pathways for potential therapeutic intervention. This investigation of global gene expression shows the importance of host immunity in the maintenance of HSV latency and provides evidence for the significant transcription activity in HSV-1 latent rabbit TG.

## Appendix 1. A group of four images generated from scanned microarrays.

To access the data, click or select the words “Appendix 1”.

Each image is generated from a scanned single 1,900 element. Each circle represents a unique spotted probe. Red genes indicate that gene expression in the rabbit HSV-1 latent trigeminal ganglia (TG) is higher than in the control (naïve), while green marks higher gene expression in the control. Yellow indicates no difference in gene expression between the latent infected treated and control TG. Black denotes absence of detectable signal

## Appendix 2. Gene expression altered in rabbit trigeminal ganglia harboring latent HSV-1

To access the data, click or select the words “Appendix 2”.

Genes whose expression was significantly (p≤0.05) altered by HSV latency. Mean log2 difference is the level of gene expression in latent trigeminal ganglia (TG) compared to naïve TG. The p-value represents the level of significance in treatment means, hit definition the name of the most homologous gene and E-value the level of confidence that the matched name is incorrect.

## Appendix 3. Genes significantly altered in rabbit HSV-1 latent trigeminal ganglia according to their Gene Ontology for biologic process.

To access the data, click or select the words “Appendix 3”.

Genes significantly altered in rabbit HSV-1 latent trigeminal ganglia according to their Gene Ontology (GO) for biologic process. The most heavily populated biologic processes included protein transport, protein biosynthesis, protein folding, protein modification, proteolysis and peptidolysis, carbohydrate metabolism and energy metabolism, cell adhesion, apoptosis, and immune and defensive responses.

## Appendix 4. Gene expression significantly altered by more than twofold in the HSV-1 latent rabbit trigeminal ganglia

To access the data, click or select the words “Appendix 4”.

Gene expression significantly (p≤0.05) altered by more than twofold in the HSV-1 latent rabbit trigeminal ganglia. Mean log2 difference is the level of gene expression in HSV-1 latent trigeminal ganglia. The p-value represents the level of significance in treatment means. Hit definition is the name of the most homologous gene. E-value is the level of confidence that the matched name is incorrect. Also listed are the Gene Ontology Consortium terms for biologic process associated with the most homologous gene.

## Appendix 5. Rabbit HSV-1 Latent trigeminal ganglia gene expression array means, their fold-changes with standard deviations and corresponding quantitative real-time polymerase chain reaction results of six selected genes

To access the data, click or select the words “Appendix 5”.

Change in gene expression of six selected genes in HSV-1 latent rabbit trigeminal ganglia were compared to naive (control) based on microarrays (Array) and quantitative real-time polymerase chain reaction. The mean log2 difference and standard error of mean log2 difference (Microarray value) for each of the selected genes is logarithmic and was obtained by the averaging the difference between microarray values for infected (L) and microarray values for naive (N) of each gene before conversion. Listed are the corresponding processed red and green signal values from the microarrays. The fold change in gene expression (Array and Quantitative real-time polymerase chain reaction) is arithmetic. Relative quantitative expression levels were determined for each gene. All results are expressed as an expression ratio of the HSV-1 latent trigeminal ganglia to naive trigeminal ganglia, normalized against housekeeping *GAPDH* expression levels using the 2^-ΔΔC^_T_ method [29]. All signal values of the arrays are distinguishable from background values, and most are high. The raw red and green signal values for the approximately 3,000 genes, 55,000 values are available on the Interdisciplinary Center for Biotechnology Research website.

## Appendix 6. Expression of 40 genes significantly downregulated in the rabbit HSV-1 latent trigeminal ganglia.

To access the data, click or select the words “Appendix 6”.

Gene expression was significantly (p≤0.05) altered by more than 2 fold in the rabbit HSV-1 latent trigeminal ganglia (TG). Forty expressed genes were significantly downregulated in the rabbit HSV-1 latent TG. Downregulation of genes in the rabbit HSV-1 latent TG were mainly in the catabolic categories of protein degradation and carbohydrate metabolism. There was no obvious pattern in gene expression that would explain the maintenance of viral latency.

## Appendix 7. Genes significantly altered in relation to the total number expressed in the Gene Ontology Categories in the HSV-1 latent rabbit trigeminal ganglia.

To access the data, click or select the words “Appendix 7”.

Genes significantly altered in relation to the total number of genes expressed in the biologic process categories in the rabbit HSV-1 latent trigeminal ganglia. Gene expressions showing the number of genes that significantly changed of the total number expressed in percentages. Shifts in regulation of the different metabolic processes show relative increases in the number of genes involved in protein metabolism compared to carbohydrate metabolism. The asterisk indicates Gene Ontology (GO), which are categories under which genes were classified.
